# Improving the quality of Health Care in the Romanian public health system - a priority in the reform process

**Published:** 2015

**Authors:** VL Purcărea, BI Coculescu, EC Coculescu

**Affiliations:** * “Carol Davila” University of Medicine and Pharmacy, Faculty of Medicine, Bucharest, Romania; ** “Titu Maiorescu” University, Faculty of Medicine, Bucharest, Romania; *** "Carol Davila” University of Medicine and Pharmacy, Faculty of Dental Medicine, Bucharest, Romania

**Keywords:** Romanian health system, health care reform, public health system

## Abstract

To meet the shortfalls caused by the economic crisis, the Romanian medical system needed an objective analysis of the quality of medical care as a whole, of the entire package of health services as well as accountable joint efforts to identify system problems and, especially, firm action without compromising resolution, regardless of any limitations or emotional picture. In addition, last but not least, the judicious use of available resources.

## Introduction

Recent studies showed that health indicators in Romania have seen a steady improvement, but it is necessary to continue the reform process so that we could reach the standards of EU countries in health.

Budgetary restrictions require the improvement of health care quality in the Romanian medical system because its absence leads to a duplication of services, and thus to an increase in spending. Unfortunately, this is still observed in health services, even if from the point of view of technical facilities, great progress has been made in the last decade of the last century, however, quality standards are not met everywhere in Romania and there is a waste of resources through duplication of investigations, analyses etc. [**1**,**2**].

## Discussion

A health system can be defined as all organizations, institutions and resources that are involved and working to promote, maintain and improve the health of the population (WHO, 2000). It includes not only hospitals, doctors, health insurance funds or ministry, but also all organizations involved in the provision and financing of services, including non-profit organizations, professional associations, private local or international providers and foundations, voluntary organizations or donors [**3**].

In principle, the development of the medical system (including the Romanian system) is primarily dependent on the level of funding and the efficiency with which this funding is used, the structure of the population and socio-economic development of the geographical area concerned, not least the attitudes and expectations of patients, which in turn translate into policies adopted by the system.

Unlike other services, health services are accessed by a large number of people, resulting in high costs of their health care.

Health insurance is an effective way by which the state can cover a significant proportion of health expenditure, but it is not the only one.

There are two types of health insurance: social insurance and private insurance. They are distinguished by two features:

• Social health insurance is mandatory, each person in the eligible group is required to join the system and pay the appropriate premium or contribution (percentage share);

• Unlike the premiums and benefits included in the private insurance, subject to a legal agreement signed jointly by the parties, those provided for by social insurance are under the law in force at that time, so that they can be changed more easily.

Health was seen until 1989 and beyond, as being an “unproductive” branch - as indeed, the entire service sector was classified as ineffective - and therefore chronically underfunded.

Since 1998, with the adoption of Law no. 145 of 24 July 1997, a new form of social insurance has been covered, that is social health insurance becoming the main system of financing health care, which provides a basic package of services for policyholders.

As a definition, social health insurance is a set of legal rules regulating medical care to employees and other persons through medical services, medicines, medical equipment and medical devices, with the aim of achieving the benefits of quality health insurance houses operating on a fund established for this purpose.

Under the legislation, they are mandatory, protecting the entire population of the country, both employees and pensioners, the unemployed and people who are not employed but are in one of the situations mentioned by Law no. 95/ 2006 on health care reform, with the subsequent completions and changes.

The global economic crisis affected the health system in our country.

Fewer financial resources, higher costs of lending to cancel reducing the investment led and will lead to further constraints in the health system (i.e. closing or merging several medical facilities, medical staff exodus due to conditions of underpay, lack of medicines and medical-pharmaceutical materials in hospitals, etc.). In addition, the current system of health insurance faces a series of problems arising from the increase in unemployment, labor migration in the EU, increasing life expectancy of pensioners, etc.

The objectives achieved by the implementation of reform in the Romanian medical system include:

• free and equal access to public health services;

• assigning new responsibilities to local government role in developing and implementing public health programs;

• precisely and transparently stating the sources of allocation of funds to the health sector;

• simplify the financial circuit of money transfer intended for health action from the state budget to local government budgets;

• increasing the role and importance of the Ministry of Health in the objectives, activities and public health structures.

Another objective that was achieved with a delay in its implementation was the integrated information system to facilitate the effective management of the existing funds.

Although health reforms have led to many organizational and functional changes, these changes have not resulted in improved health status of the population in Romania. Instead, there is an increasing trend of mortality and an increase in the number of deaths from preventable causes of death. According to the Report of the Presidential Commission for the analysis and public health policy elaboration (“A medical system centered on the needs of citizens”), in terms of health, Romania’s population presents some of the worst indicators in the whole European area, not only at the EU level. One of the indicators drawing attention is life expectancy (71.7 years) which, although presents a slight improvement, continues to be among the lowest in the European Region [**4**].

The increase in the percentage from year to year health system costs relative to gross domestic product (GDP) requires precise valuation requirements of the care system both in terms of cost and in terms of clinical efficacy.

The need for reform of the health system has manifested especially in recent years and is not only specific to Romania. It is a global process that aims to decrease the gap between the population’s health expectations and the response provided by the medical system to these expectations.

The main conditions which must be fulfilled by a performing health system refer to: overall coverage; prompt accessibility; relevance to needs; equity; choice; effectiveness; high efficiency; broad social accessibility; State responsibility to public health. It is obvious that a real health system cannot have all these qualities and, even if it meets these requirements, it cannot be maintained in this state, being in a permanent change due to socio-economic conditions and the increasing expectations of patients [**5**,**6**].

Reforming healthcare systems in the European Union had the following priority objectives:

• efficient resource management and planning;

• decentralization in the health care systems;

• wide accessibility of the population to health services;

• improving community health services;

• priority for preventive health services based on prevalent risk factors;

• improving medical staff

The introduction in the medical field of this wide-range reform led to the functioning of health insurance, decentralization of services with emphasis on the role of outpatient medical services, amending the medical legislative framework, defining and enforcement of quality norms in health care, and so on.

The main levers of intervention on the health care system are:

• Financing: mechanism for generating funds to support the health sector activity;

The main sources of funds can be taxes, social security contributions, private insurance premiums or direct payments. There can be other sources such as external financing or a form called Community funding. Most systems are mixed, so it is impossible to locate a country entirely within a particular category.

• Payment: the ways in which the money raised by funding is transferred to individuals and organizations in the health sector;

Institutions (mainly hospitals) can be paid in several ways: on admission, per day, per service or an overall budget. Practitioners can also be paid per capita for those they have in care, per case, per service or salary. Each of these inserts certain incentive effects.

• Organization: macrostructure and microstructure.

Macrostructure refers to the set of organizations, their functional roles, their leadership and their sources of funding. Microstructure refers to what happens within the organization; that is the way it influences the kind of people it recruits, the incentives they offer and structure of authority, responsibility and decision-making, the way the organization responds to the incentives generated by funding and payment systems.

• Regulatory: mechanisms of exercising constraints on behavior in healthcare organizations.

They can be focused on entering the system, on price, quality and relationships between seller and buyer. The regulation is one of the possible reactions of society when the organizations’ response to the incentives they have is not sanctioned by society. 

• Social Marketing: systematic influence on attitudes and practices, using techniques inspired from commercial marketing, pursuing to obtain benefits for the target audience or society in general [**3**].

Currently, the medical system in Romania is facing its underfunding, requiring inter alia substantial investment in infrastructure to support public health institutions.

One of the proposals for reform aimed for the package of basic services to be redefined in order to cover only the social component, but at the same time be in conjunction with the existing financial resources in each fiscal year:

- Developing complementary health insurance systems, private insurance, which will supplement the basic package of services;

- Non-discriminatory treatment to insurance and health care providers;

- Regulation of tariffs so that they are closely related to each type of medical benefit basis [**6**,**7**].

Other solutions aimed at creating a modern medical system by introducing the principle of competition between health service providers for an efficient use of the existing funds, the state adopting a neutral stance towards all suppliers and ensuring permanent quality control services to policyholders, focusing on the creation of specific departments at the National House of Health Insurance (NHHI) and the territorial health insurance funds.

Any reform of the health system in Romania has to cover two major components:

• institutional framework and

• medical services

and within these, the mandatory social health insurances in accordance to market economy and EU practices play an important role. The main objective of the reform should be revenue growth in social health insurance system, for it to become truly effective [**8**].

However, it should be noted that the collection of funds is only part of a medical system; their methods of distribution – i.e. management of these funds to the suppliers - are, in fact, those determining the system performance [**9**].

Moreover, in order to use the available resources efficiently, it is necessary to introduce new, clearer and more efficient healthcare politics, new working tools and financing mechanisms, which will bring an effective improvement in both the allocative and the technical efficiency. The allocative efficiency imposes the use of the available limited resources in certain directions, which will produce a maximum level of wellbeing, meaning a maximization of the impact on the health state. The technical efficiency represents the way the resources are used at the level of the providers in order to achieve the expected results. The more less resources are used to achieve the expected results, the more the provider’s efficiency raises [**10**-**13**]. 

The social health insurance system shall operate based on universal principles: solidarity (equal percentage contribution from insured persons’ incomes), equity, subsidiarity. Private health insurance will only have to cover the pay for the difference between the amounts covered by social health insurance and the amount of benefits each medical and pharmaceutical services provider offers, as well as the pay for those medical services that are not covered by the social health insurance. An indirect effect of the reform could be the reduction of informal payments or gifts that patients give to medical personnel.

The demarcation of clear coordinated responsibilities between the main institutions involved in the health system reform and functioning: Ministry of Public Health, the County and Bucharest Public Health, the National Health Insurance and county health insurance houses, College of Physicians in Romania, District Colleges of Physicians, and of course, health care providers should ensure the development and implementation of clear rules and effective balanced integration of all components of the public health system. In parallel, the national legislation must provide the independent functioning of the National House of Health Insurance, without political ownership of its activity. In addition, according to the same legislative regulation, it is necessary to introduce mandatory private health insurance, an important factor in increasing the quality of health insurance provided to policyholders.

The efficiency of these elements of reform and an increase in the control system computerization should also be provided to carry out the correlation between the medical information management system and information system in health insurance. In addition, if properly placed in health care, competition can reduce costs, improve performance and create better medical outcomes for patients [**10**].

Finally, it is worth noting that the new package of health services [**14**] is focused on a new concept, which emphasizes preventive medicine. Therefore, from this perspective, a rethinking of the medical practices in schools would be beneficial, regarding the place and role they should play within the national broader prevention program. This, in a context where, by emergency ordinance, medical practice in school was subordinated to local authorities, today we are faced with an alarming situation: in many schools medical offices have been dissolved, either from lack of funds, or - where they exist and are properly equipped - they cannot work due to blocked positions for medical doctors [**11**].

## Conclusions

In countries with developed health systems, it has been proved that competition among medical organizations within the health care system is not able to drive value for the patient, so the change is intended to become more efficient in order to achieve greater satisfaction to the patient. There is hope that the governments from developed countries could achieve the implementation and development of key health policies, with particular emphasis on obtaining information on results, and removal of impediments that may arise in the competitive environment. 

The success of healthcare organizations is subject to accurate knowledge of the expectations of consumers / clients / patients, government requirements and competitors’ criticisms, and society’s in general, in terms of social responsibility. Respect for the patients’ rights is one of the most important issues of social responsibility in the protection of the beneficiaries of health services aiming at the achievement of objectives, including performance and reliability of the service, provision of relevant information on the services offered, etc.

In the process of establishing a direct relationship with the clients, the healthcare organizations must revise their offers, cost structures and competitive platforms in order to line up to the short chain of information, to the continually changing requests and the clients’ behavior. The current technology represents the connection between sources, services, networks and clients. In addition, as the service becomes more comfortable, the competition increases and the differentiation becomes a vital issue. While the services market is in a continuous movement and transformation, the healthcare organizations should understand this information and the working processes and should analyze each level of the process. In other words, the processes should have a more specific and more attentively administered character than before. They must synchronize closely with the ones of the other organizations but also with the clients whom they could collaborate with or even take part in the process of producing the end result. Due to the changes in the infrastructure and at technology level, the competition and the healthcare industry are restructuring, the healthcare services organizations must test their systems constantly if they wish to negotiate these changes on long-term [**15**,**16**].

In addition, as stated before, the profoundness of the process of changes in the healthcare field, its dimension and dynamic, do not depend on the political will or the punctual subjective aspirations, but, firstly, on the existence and the sufficiency of the necessary objective conditions [**10**]. 
